# Chemokine expression in the early response to injury in human airway epithelial cells

**DOI:** 10.1371/journal.pone.0193334

**Published:** 2018-03-13

**Authors:** Bingqing Xie, Bharathi Laxman, Somaye Hashemifar, Randi Stern, T. Conrad Gilliam, Natalia Maltsev, Steven R. White

**Affiliations:** 1 Department of Human Genetics, University of Chicago, Chicago, IL, United States of America; 2 Illinois Institute of Technology, Chicago, IL, United States of America; 3 Department of Medicine, University of Chicago, Chicago, IL, United States of America; 4 Toyota Technological Institute at Chicago, Chicago, IL, United States of America; National Jewish Health, UNITED STATES

## Abstract

Basal airway epithelial cells (AEC) constitute stem/progenitor cells within the central airways and respond to mucosal injury in an ordered sequence of spreading, migration, proliferation, and differentiation to needed cell types. However, dynamic gene transcription in the early events after mucosal injury has not been studied in AEC. We examined gene expression using microarrays following mechanical injury (MI) in primary human AEC grown in submersion culture to generate basal cells and in the air-liquid interface to generate differentiated AEC (dAEC) that include goblet and ciliated cells. A select group of ~150 genes was in differential expression (DE) within 2–24 hr after MI, and enrichment analysis of these genes showed over-representation of functional categories related to inflammatory cytokines and chemokines. Network-based gene prioritization and network reconstruction using the PINTA heat kernel diffusion algorithm demonstrated highly connected networks that were richer in differentiated AEC compared to basal cells. Similar experiments done in basal AEC collected from asthmatic donor lungs demonstrated substantial changes in DE genes and functional categories related to inflammation compared to basal AEC from normal donors. In dAEC, similar but more modest differences were observed. We demonstrate that the AEC transcription signature after MI identifies genes and pathways that are important to the initiation and perpetuation of airway mucosal inflammation. Gene expression occurs quickly after injury and is more profound in differentiated AEC, and is altered in AEC from asthmatic airways. Our data suggest that the early response to injury is substantially different in asthmatic airways, particularly in basal airway epithelial cells.

## Introduction

The epithelium lining human lung airways serves as a primary defense interface against particulates, inhaled pollutants, pathogens, and xenobiotics. Basal airway epithelial cells (AEC) comprise approximately 30 percent of the epithelium in the major airways. These cells operate as the progenitors for ciliated and goblet columnar cells within the airway [1–3], especially after mucosal injury [1, 4, 5]. A baseline transcriptome of basal AECs is clearly distinct from the differentiated AEC (dAEC) [6, 7], and the dAEC transcriptome in culture conditions more closely resembles that of an in vivo airway epithelium [8].

Airways diseases, including asthma, substantially alter the epithelial response to injury [9, 10]. Ordered gene expression in mucosal repair after injury seen in normal airways may be dysfunctional in asthma, and injury culture models of asthmatic AEC demonstrate a shift towards the expression of genes associated with disordered repair and remodeling [11–13]. Normal epithelial cells release a number of cytokines and chemokines following direct physical injury or exposure to environmental stressors and bacterial or viral pathogens [9]. However, the inflammatory response to injury in asthmatic cells remains enigmatic. Several studies have reported significant differences observed in airway epithelial cells recovered from asthmatic subjects as compared to normal AEC. Moreover, asthmatic AEC may retain certain of these abnormalities even when in culture [14–20], such as mitotic dys-synchrony and increased secretion of inflammatory cytokines such as IL-6, IL-13, and TGF-β1 after mechanical injury [12, 19].

Despite the importance of understanding how an asthmatic epithelium releases inflammatory mediators in response to environmental perturbation, little is known about the activation of gene networks in the first hours after injury. These networks of the early-responder genes are essential for shaping the immediate epithelial cell response. Heguy et al. [21] examined gene expression seven and fourteen days after abrasive injury *in vivo* by repeated sampling of the same location in an airway via bronchoscopy, and demonstrated a distinctive ‘repair transcriptome’ at seven days that was dominated by cell cycle, signal transduction, metabolism, and transport genes. The transcriptome reverted more closely to a resting expression profile by the fourteenth day after the injury. However, these time points do not characterize the immediate (within hours) changes in the transcriptome after injury.

To address the questions related to the early events that occur after injury, we examined epithelial cell expression in a well-established mechanical injury model in both normal and asthmatic AEC grown under two distinct conditions: (1) in submersion culture with uniformly basal AEC phenotype, and (2) in air-liquid interface (ALI) culture that generates a more differentiated AEC (dAEC) phenotype seen in a homeostatic airway.

Our data demonstrated the expression of pro-inflammatory cytokines and chemokines genes within hours of injury in normal but not asthmatic cells. Clear differences were also seen between the two cell phenotypes with greater expression of inflammatory cytokines after injury noted in differentiated AEC. Furthermore, the response to MI of asthmatic AEC in either culture environment responded differently compared to normal AEC and expressed genes that were related to cell cycle and proliferation, programmed cell death, and antimicrobial activity as opposed to cytokines and chemokines. These data suggest that epithelial cells can within hours express genes that encode inflammatory mediators and that these may differ qualitatively in asthmatic versus normal epithelial cells.

## Materials and methods

### Cell culture

Primary human tracheobronchial epithelial cells were collected from lungs provided by the Gift of Hope Organ & Tissue Donor Network, Itasca, IL, through the generous gift of donor families. Donors had previously provided consent for the use of discarded tissue in research to the Gift of Hope. These lungs had been collected for use in lung transplantation but rejected for use, and were considered to be exempt from IRB regulation by the University of Chicago IRB (S1 Letter). In considering this exemption, the IRB noted that the rules of the Office of Human Research Protection (45 CFR46.102 (f)) did not apply, as the donors were deceased and that we did not obtain information about or samples from living individuals, so that the research described in this manuscript did not constitute engagement in human subjects research. No donated tissue and organs came from any vulnerable populations.

Lungs were collected from two groups of donors: those without known lung disease, and those with a history of chronic asthma. For the latter group, no specific information generally was available concerning disease severity, duration, or treatment. No other clinical or identifying information provided by the Gift of Hope was used or is disclosed in this study.

To obtain either basal cell or differentiated cell phenotypes, AEC were grown in submersion or ALI culture (21 days) respectively [17, 22, 23]. Passage 1 cells used in ALI culture were seeded at 1.0 x 10^5^ cells onto collagen IV-coated, 12.5 mm diameter Millicell CM culture inserts (pore size 0.4 μm) (Millipore, Inc.). Cells were grown to 80% confluence, the apical medium was removed and cells were fed via the basolateral side every other day to day 21. Passage 1 cells used in submersion culture were seeded similarly onto collagen IV-coated plastic wells, 12.5 mm diameter, and grown to 100% confluence. Cell morphology was confirmed in cultures from each donor using immunostaining antibodies directed against cytokeratin 5 (CK5) to mark basal cells (either goat IgG sc-17090 or mouse IgG1 sc-32721, both used at 1:250 dilution Santa Cruz) [24, 25], alpha-acetylated tubulin (AA-tubulin) to mark ciliated cells (mouse IgG2b #32–2700, 1:500 dilution, Zymed) [26, 27] or MUC5AC (either goat IgG sc-16910 or rabbit IgG sc20118, both used at 1:250 dilution, Santa Cruz), to mark mucoid cells [28, 29]. Cells were first fixed in 100% ethanol at 4°C for 30 minutes, after which cells were washed, and primary antibodies were incubated overnight at 4°C. Secondary antibodies directed against each with non-overlapping conjugated fluoroprobes then were added (2 mg/ml each as required) at 20°C for 1 hr: donkey-anti rabbit IgG conjugated with Alexa Fluor 594; donkey-anti mouse IgG (H+L) conjugated with Alexa Fluor 488; donkey anti-goat IgG (H+L) conjugated with Alexa Fluor 594; donkey anti-goat IgG (H+L) conjugated with Alexa Fluor 488; and donkey anti-mouse IgG (H+L) conjugated with Alexa Fluor 594 (all Invitrogen). Nuclei were labeled with Hoechst 33258. Cells were imaged using an Olympus DSU disk scanning confocal system on an Olympus IX81 microscope platform equipped with a Photometrics Evolve- back-thinned CCD camera and UPlan Apo 40x or 60x objectives. Images were assembled from z-stacks using *ImageJ* software (Wayne Rasband, National Institutes of Health, Bethesda, MD).

### Mechanical injury experiments

Cells grown to confluence in either culture system were injured using a mechanical rake to ensure a large number of wound edges [30], then incubated for up to 24 hr. Control cells without mechanical injury (MI) were collected at the same time intervals.

### Microarray processing

Total RNA was extracted from cells using QIAzol lysis reagent followed by RNeasy mini kits (Qiagen, Inc., Valencia, CA). Gene expression profiling was assessed using HumanHT-12 v4 expression BeadChips (Illumina, San Diego, CA) that included probes for more than 47,000 genome-wide transcripts.

### Differential expression analysis

We first grouped the expression data into 12 groups, each group with only one variant of injury versus no-injury. The 12 groups contain each time point (2, 8, and 24 hour), two culture conditions (submersion and ALI) and two disease states (asthma and normal). Two major methods of analysis, weighted gene correlation network analysis (WGCNA) and differential expression based analysis, were applied to these groups. For microarrays passing initial quality control, expression levels were extracted using Limma after background subtraction and quantile normalization using the method of Xie et al. [31]. Significant DE was determined following correction for multiple testing and false discovery by the method of Benjamini and Hochberg (23) using Limma [32]. Genes in DE between ALI and submersion conditions (absolute FC ≥ 1.5, corrected P value < 0.05) for every time point under consideration were extracted.

Gene expression pattern analysis was performed to identify the sets of genes differentially expressed under each culture condition over time [33]. Heat-maps and a linear plot for the significantly differentially expressed genes were generated to indicate the levels of expression and the relationship among the genes at different times and conditions. Enrichment analysis for identification of GO categories, diseases, phenotypes, pathways, and other features over-represented in the sets of differentially expressed genes, was performed using online analytical tools available in the Lynx integrated system [34]. Statistical significance was estimated using Bayes factor and P-value calculated for each category under consideration. Bonferroni correction was applied to the results of analyses to correct for multiple testing. MIAME-compliant raw data has been deposited in the Gene Expression Omnibus (GEO) site (http://www.ncbi.nlm.nih.gov/geo) (accession numbers GSE 59128 and GSE109170).

### Network-based gene prioritization

Genes differentially-expressed under each condition were submitted to the Lynx/PINTA server. The PINTA [35] heat kernel diffusion algorithm was used to generate a gene association score that estimates the strength of functional associations between the differentially expressed genes and prediction of additional genes potentially involved in the inflammation/wound healing response under each condition. The original heat kernel diffusion algorithm from the PINTA tool was modified to accommodate weighted data types. The STRING platform was used as the underlying genome wide functional association network for gene prioritization and network reconstruction [36, 37]. Two iterations for network diffusion; 0.5 as the diffusion value, and 5,000 network randomizations for estimating p-values, were used as parameters for the heat kernel diffusion algorithm. Bonferroni correction was applied to the results of analyses to correct for multiple testing. Genes with the score ≥ 0.03 were extracted for each condition. The resulted patterns and network-based predictions were visualized by the STRING webserver using default settings for confidence, evidence and molecular action [36].

### Weighted gene co-expression network analysis

Systems level analysis of cellular responses was done by WGCNA [38, 39]. This analysis was performed separately for each group as well for the whole experiment to avoid the dilution of the co-expression patterns by mixing the experimental conditions. The initial step included the reconstruction of the co-expression network based on Pearson correlation. The threshold for this network was determined by the connectivity and degree distribution. Module detection was performed on the resulting co-expression network by hierarchical clustering. Each module contained subsets of genes in the input expression files; modules did not overlap with each other. The genes within each module were considered to be co-expressed. The modules then were evaluated by whether the overall expression of genes within the module correlated, positively or negatively, with injury/no-injury as the condition variable. An estimated P-value was generated for each module to estimate the significance of the corresponding correlation. Functional enrichment analysis then extracted significant categories from each module which was compared across the groups.

## Results

### Epithelial transcriptomes of normal un-injured basal AEC and dAEC are different under standard quiescent culture

**Fig 1:**
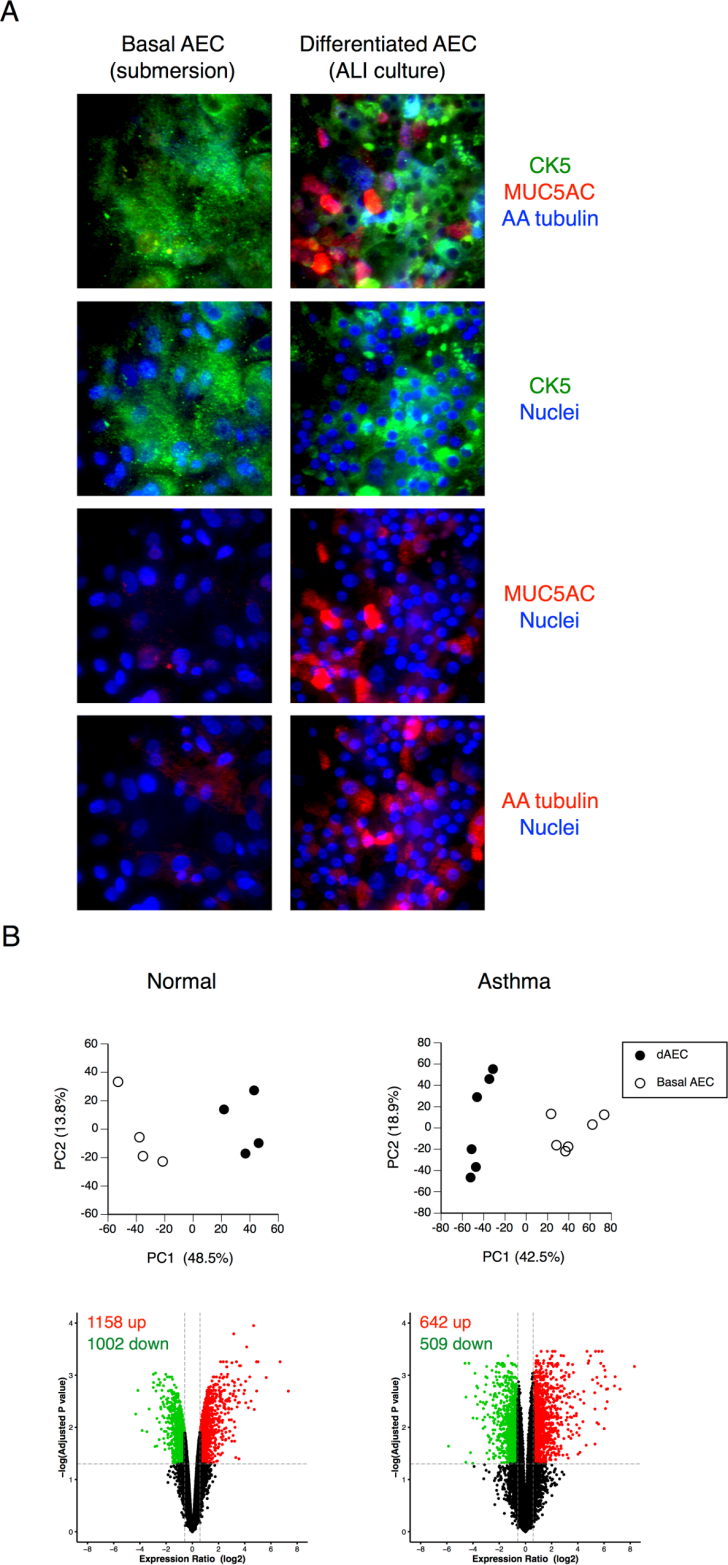
Demonstration of airway epithelial cell differentiation. A. Confocal immunofluorescence of basal and differentiated (dAEC) to demonstrate markers of differentiation in the latter. The presence of MUC5AC indicated differentiation into goblet cells, and the presence of alpha-acetylated tubulin indicated differentiation into ciliated cells. The presence of CK5 indicated the presence of basal cells in both culture conditions. No significant differentiation into goblet or ciliated cells types is seen in cells grown in submersion culture. B. Principal component analysis and volcano plots in AEC from normal or asthmatic donor lungs to demonstrate differential gene expression in the resting (without mechanical injury) state (N = 4 in each group) between dAEC and basal AEC, using all expressed gene probe sets (n = 35,530 for normal cells and n = 41,733 for asthmatic cells) as an input dataset. For each, the number of up- and down-regulated probes (≥1.5 fold change, vertical dashed lines, adjusted P < 0.05, horizontal dashed line) is provided.

Basal AEC grown in submersion culture expressed cytokeratin 5 (KRT5) and did not express significant quantities of either gel-forming mucin 5AC (MUC5AC), characteristic of goblet epithelial cells, or of alpha-acetylated tubulin, characteristic of ciliated epithelial cells, as reported by the previous studies [6, 22]. As expected, dAEC culture demonstrated abundant expression of both MUC5AC and alpha-acetylated tubulin in addition to cytokeratin 5, indicating the presence of goblet and ciliated cells, respectively (Fig 1A). The whole genome transcriptomes of normal dAEC and basal AEC from four donor lungs also demonstrated significant differences as assessed by both PCA and volcano plots of DE (Fig 1B) as expected [6] for differences in cell type and development. Table A in S1 File provides a list of genes in DE between the two culture conditions.

The enrichment analysis of the genes differentially expressed (logFC>1.3, FDR-corrected P-value < 0.05) between the normal uninjured basal AEC and differentiated dAEC was performed using both the Lynx [34, 40] and ToppGene [41] bioinformatics platforms. There were substantial differences between these two culture conditions at 8 hr after MI. The genes highly expressed in the basal AEC cells were predominantly involved in keratinization (GO:0031424) and cell cycle (GO:0007049). However, the genes highly expressed in the dAEC cells at the same time point were enriched in the categories related to cytokine activity (GO:0005125), extracellular space (GO:0005615), and interferon signaling (GO:0060333) (Table B in S1 File).

### Epithelial transcriptomes in normal basal and dAEC differ after mechanical injury

**Fig 2:**
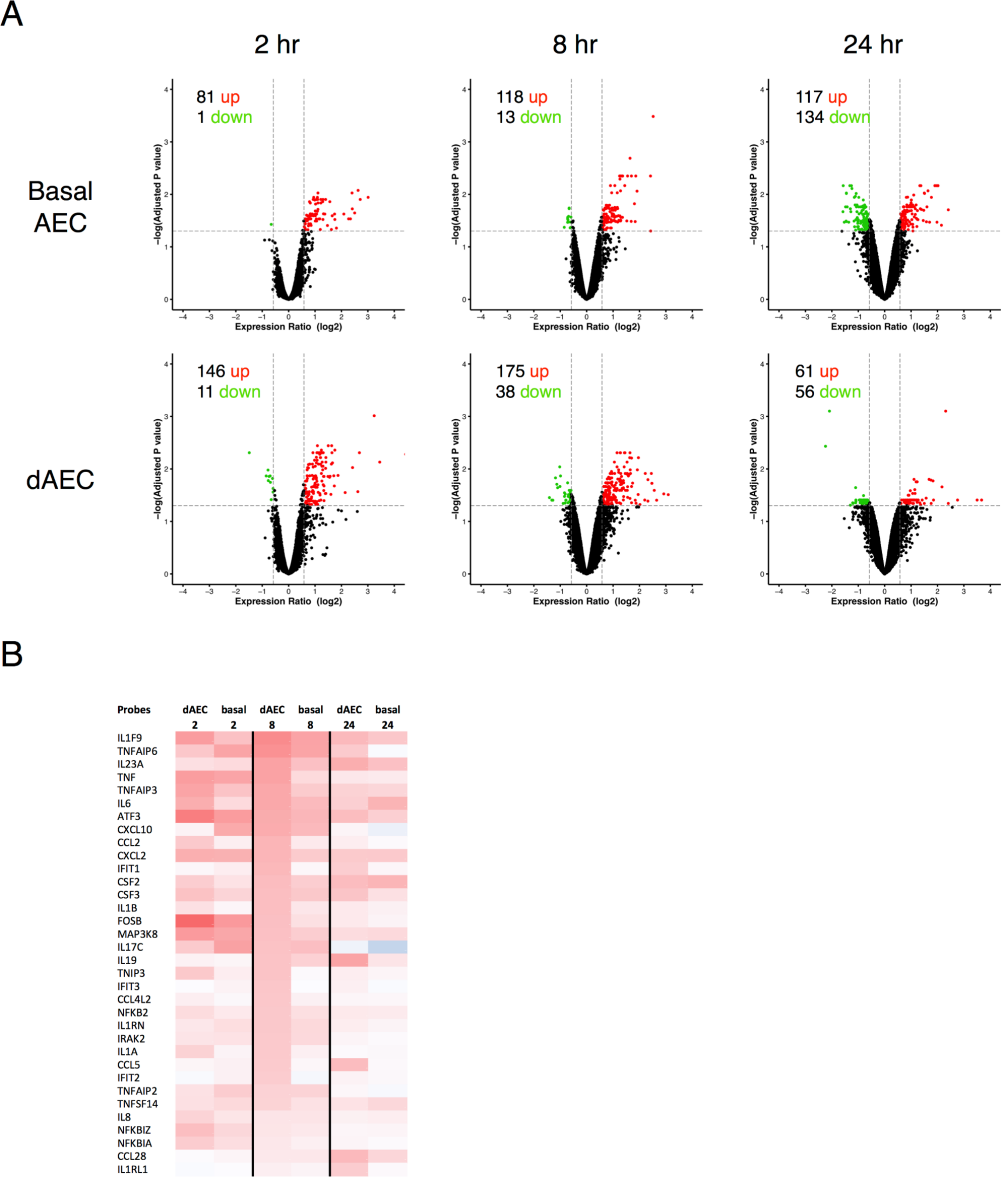
Normal basal and differentiated airway epithelial cells differ in gene expression patterns after mechanical injury. A. Volcano plots in AEC from normal donor lungs to demonstrate differential expression in probes in the resting (without mechanical injury) state (N = 4 in each group) between differentiated (dAEC) and basal AEC, using all expressed gene probe sets (n = 35,530) as an input dataset. For each, the number of up- and down-regulated probes (≥1.5 fold change, vertical dashed lines, adjusted P < 0.05, horizontal dashed line) is provided. B. Differential expression for selected gene probes involved in inflammation and inflammation signaling at 2–24 hr after injury versus non-injured cells is provided as a heat map.

Mechanical injury to normal AEC and dAEC was done using a rake that generated a substantial number of cells at wound edges [30]. The analysis of differential expression of genes in both normal differentiated and basal AEC at 2, 8 and 24 hours after MI demonstrated (Fig 2A) a significant number of DE genes. Table C in S1 File lists 87 genes of interest to inflammation; as shown in Fig 2B, a number of inflammatory cytokine and chemokine ligands, and key signaling intermediates for these ligands, were up-regulated in both differentiated and basal AEC 2 to 24 hr hours after mechanical injury.

Tables D—G in S1 File list genes related to cell cycle, differentiation, proliferation, and cell migration. Using one-tailed paired t-tests, we examined gene expression after injury in differentiated AEC as well as basal AEC for genes associated with cell cycle and differentiation. In both culture conditions at 8 hr after MI, there were genes in significant DE for both cell cycle (P < 0.01) and differentiation (P < 0.02) related genes. Therefore, genes involved in these two biological process have significant (P < 0.05) difference between dAEC and basal AEC as expected.

Enrichment analysis was performed for identification of the functional categories overrepresented in the sets of the genes differentially expressed in normal vs injured AEC or dAEC cells. Table 1 presents the results of the enrichment analysis of the DE genes in normal dAEC and AEC 8 hr after injury. Both basal AEC and dAEC demonstrated significant activation of molecular pathways involved in cytokine activity (GO:0001817, REACT_75790), inflammation (GO:0006954), and to defense responses against bacteria (GO:0002684). Activation of the response to injury was observed in both cell culture conditions but different sets of genes were activated in the two different cell types (Table 1).

**Table 1:** Enrichment analysis of the DE genes in normal basal AEC and dAEC 8 hours after mechanical injury.

Feature ID Name	Cell Type	Genes	P Value	Bayes
GO:0001817 regulation of cytokine production	Normal dAEC	ADAM8 CCL2 CD83 CSF2 CYP1B1 HERC5 IFIH1 IKBKE IL1A IL1B IL23A IL36G IL6 ISL1 KLF4 MAP2K3 NFKB2 PDE4B PELI1 PTGS2 STAT5A TICAM1 TNF TNFAIP3 ZBP1 ZC3H12A ZP3	1.88E-14	26.558
	Normal Basal AEC	ATF4 CSF2 HMOX1 IDO1 IL17C IL1B IL23A IL36G IL6 IRAK3 KLF4 NFKB2 TICAM1 TNF TNFAIP3 ZC3H12A	6.79E-09	14.25
GO:0006954 inflammatory response	Normal dAEC	ADAM8 ADORA2A CCL2 CXCL10 IL1A IL1B IL23A IL36G IL6 IRAK2 MAP2K3 NFKB2 NOS2 PLA2G4C PTGS2 TICAM1 TNF TNFAIP3 TNFAIP6 TNIP1 TNIP3 VNN1	1.02E-13	24.842
	Normal Basal AEC	CXCL10 CXCL2 HDAC9 IL17C IL19 IL1B IL23A IL36G IL6 IRAK2 NFKB2 TICAM1 TNF TNFAIP3 TNFAIP6 TNIP1	1.77E-11	20.148
GO:0043067 regulation of programmed cell death	Normal dAEC	ADAM8 ADORA2A ATF3 BCL11B BIRC3 CARD10 CCL2 CLCF1 CSF2 CYP1B1 CYR61 DEPTOR EDN1 GDF15 ICAM1 IFIH1 IFIT2 IFIT3 IL1A IL1B IL1RN IL6 ISL1 JUN KLF4 MAP2K3 MAP3K8 OSGIN1 PIM1 PIM3 PLAUR PMAIP1 PTGS2 SNCA SOD2 SQSTM1 STAT5A TICAM1 TNF TNFAIP3 TP53BP2 VAV3 VNN1	9.00E-14	25.099
	Normal Basal AEC	ATF3 ATF4 BID BIRC3 CSF2 GDF15 HMOX1 IDO1 IL19 IL1B IL1RN IL6 IRS2 JUN KLF4 MAP3K8 OSGIN1 PLAUR PMAIP1 RHOB SGK1 SIRT1 SOD2 TICAM1 TNF TNFAIP3 TP53BP2	1.98E-09	15.571
GO:0080134 regulation of response to stress	Normal dAEC	ADAM8 ADORA2A BIRC3 C1QTNF1 DUOXA2 EDN1 HBEGF HERC5 IFIH1 IFIT1 IKBKE IL1A IL1B IL1RN IL23A IL6 IRAK2 ISL1 ITGAM ITPKC JUN KLF4 MAP2K3 NFKB2 PELI1 PLAU PLAUR PMAIP1 PPP1R15A PTGS2 SASH1 SESN3 SH2D1B SLC7A2 SNCA SOD2 SQSTM1 STAT5A TICAM1 TNF TNFAIP3 TNFAIP6 TNIP1 TNIP3 VNN1 ZBP1 ZP3	2.08E-18	35.719
	Normal Basal AEC	ATF4 BIRC3 C1QTNF1 CFB IDO1 IL17C IL1B IL1RN IL23A IL6 IRAK2 IRAK3 JUN KLF4 NFKB2 PLAUR PMAIP1 PPP1R15A SIRT1 SLC7A2 SOD2 TICAM1 TNF TNFAIP3 TNFAIP6 TNIP1	9.32E-10	16.31
GO:1903034 regulation of response to wounding	Normal dAEC	ADAM8 ADORA2A BIRC3 C1QTNF1 DUOXA2 EDN1 HBEGF IL1B IL23A IL6 ISL1 KLF4 PLAU PLAUR PTGS2 SLC7A2 STAT5A TNF TNFAIP3 TNFAIP6 TNIP1 ZP3	6.87E-13	22.948
	Normal Basal AEC	BIRC3 C1QTNF1 CFB IDO1 IL17C IL1B IL23A IL6 KLF4 PLAUR SLC7A2 TNF TNFAIP3 TNFAIP6 TNIP1	7.26E-10	16.454
KEGG:hsa04668 TNF signaling pathway	Normal dAEC	BIRC3 CCL2 CSF2 CXCL10 EDN1 ICAM1 IL1B IL6 JUN MAP2K3 MAP3K8 PTGS2 TNF TNFAIP3	5.39E-13	23.123
	Normal Basal AEC	ATF4 BIRC3 CSF2 CXCL10 CXCL2 IL1B IL6 JUN MAP3K8 TNF TNFAIP3	1.23E-11	20.465
REACT_75790 Cytokine Signaling in Immune system	Normal dAEC	CSF2 HERC5 ICAM1 IFIT1 IFIT2 IFIT3 IL1A IL1B IL1RN IL6 IL7R IRAK2 MAP3K8 MX1 MX2 NFKB2 OAS2 OAS3 OASL PELI1 SQSTM1 STAT5A TRIM31	2.09E-15	28.718
	Normal Basal AEC	CSF2 IL1B IL1RN IL6 IRAK2 IRAK3 IRS2 MAP3K8 NFKB2 TRIM31 TRIM6	6.29E-07	9.708
WP1449 Regulation of toll-like receptor signaling pathway	Normal dAEC	CXCL10 IKBKE IL1B IL6 IRAK2 JUN MAP2K3 MAP3K8 NFKB2 PELI1 SQSTM1 TICAM1 TNF TNFAIP3	3.26E-11	19.039
	Normal Basal AEC	CXCL10 IL1B IL6 IRAK2 IRAK3 JUN MAP3K8 NFKB2 TICAM1 TNF TNFAIP3	3.13E-10	17.244

Systems level analysis of these responses was characterized using weighted gene co-expression network analysis (WGCNA) [38, 39]. The analysis was done for each time point and cell type using only significantly differentially expressed genes (FC > 1.5, adjusted P value < 0.05) to identify gene functional modules specific for the condition under observation. An absolute correlation coefficient > 0.8 and adjusted P value < 0.05 was required for significance. Table H in S1 File presents the statistically significant modules characteristic for each condition. The significant modules detected at 8 hr after MI were enriched for cytokine activity (GO:0005125), interleukin-1 receptor binding (GO:0005149), and inflammatory responses (GO:0006954) in both differentiated and basal AEC, while differentiated AEC also showed enrichment for toll-like receptor pathways (GO:0034142, GO:0002224, GO:0034138) (Table I in S1 File). A greater number of GO functional categories were identified as significant for dAEC as compared to basal AEC.

**Fig 3:**
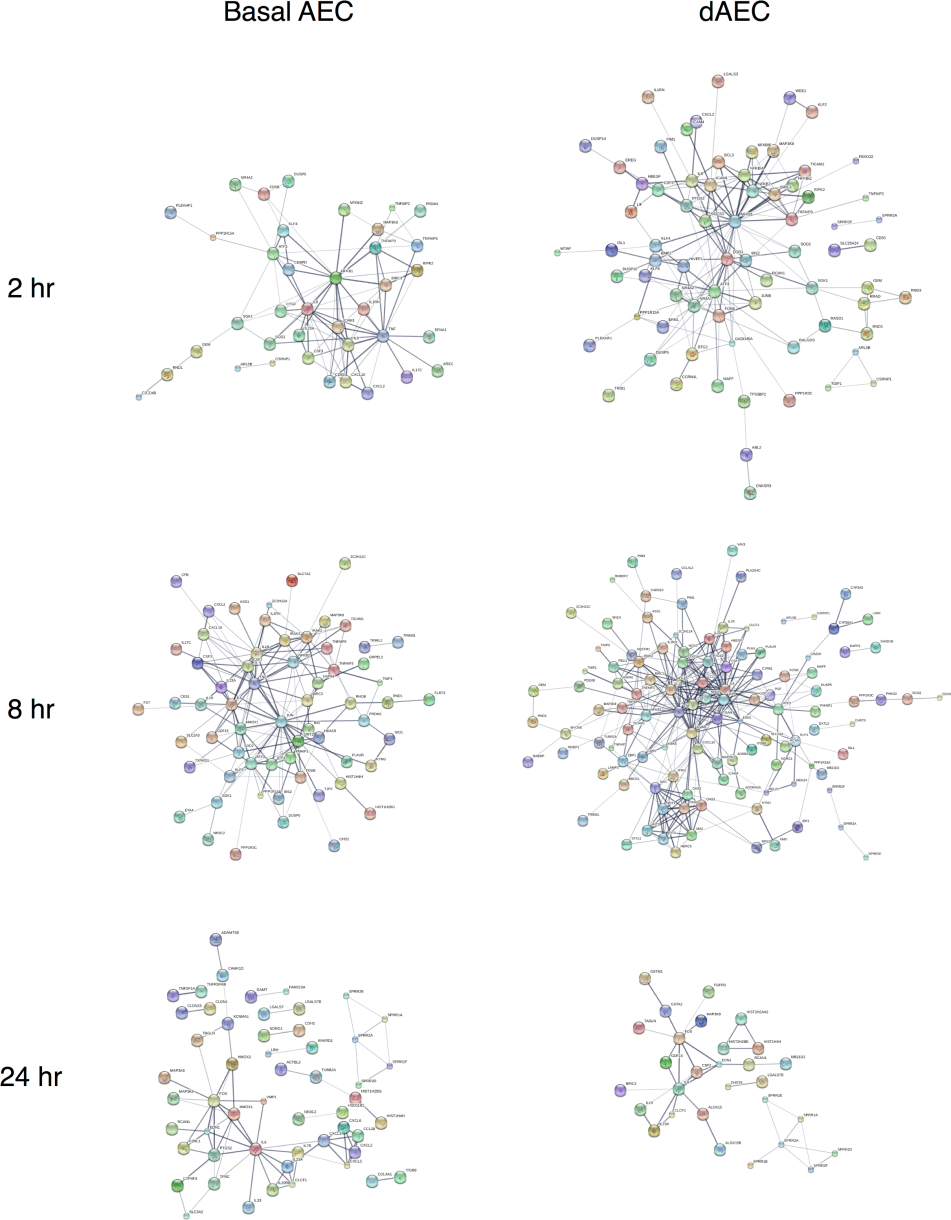
Reconstruction of molecular networks for normal basal and differentiated AEC after mechanical injury. String networks generated from WGCNA modules using the genes in significant DE at 2–24 hr after injury for each cell type to demonstrate functional relationships in molecular networks. Highly connected network activation was richer and more substantial in differentiated (dAEC) versus basal AEC, particularly at 2 and 8 hr after MI, but less so at 24 hr. Probe labels are as shown.

Reconstruction of the molecular networks using DE genes (P < 0.05 and FC > 1.5) as seed genes for dAEC and AEC at each time point are presented in Fig 3. There was an increase in membership and complexity of the anti-inflammatory response at 8 and 24 hr, as compared to 2 hr, after MI for both basal and differentiated AEC. However, at 8 hr after MI, the DE genes network for differentiated AEC (86 nodes) are more complex than their basal AEC counterparts (47 edges) (Fig 3 and Table J in S1 File). Especially at 8 hr after MI, the differentiated AEC network contains twice as many edges as basal AEC network and the differentiated AEC network has a higher average degree (8.047) than basal AEC network (6.809). These data indicated that in the immediate hours after injury, both differentiated and basal AEC elicited gene expression that is related to a significant inflammatory and pro-defense response that in differentiated AEC is more intense.

### Epithelial transcriptomes in basal and dAEC differ after mechanical injury specific to asthmatic cells

As with cells collected from normal donor lungs, transcriptomes of dAEC and basal AEC collected from asthmatic donor lungs demonstrated appreciable numbers of ciliated and goblet cells when grown in air-liquid interface and a significant number of up-regulated and down-regulated genes illustrated by volcano plot (FC ≥ 1.5 and corrected P value [FDR] < 0.05) (Table A in S1 File).

**Fig 4:**
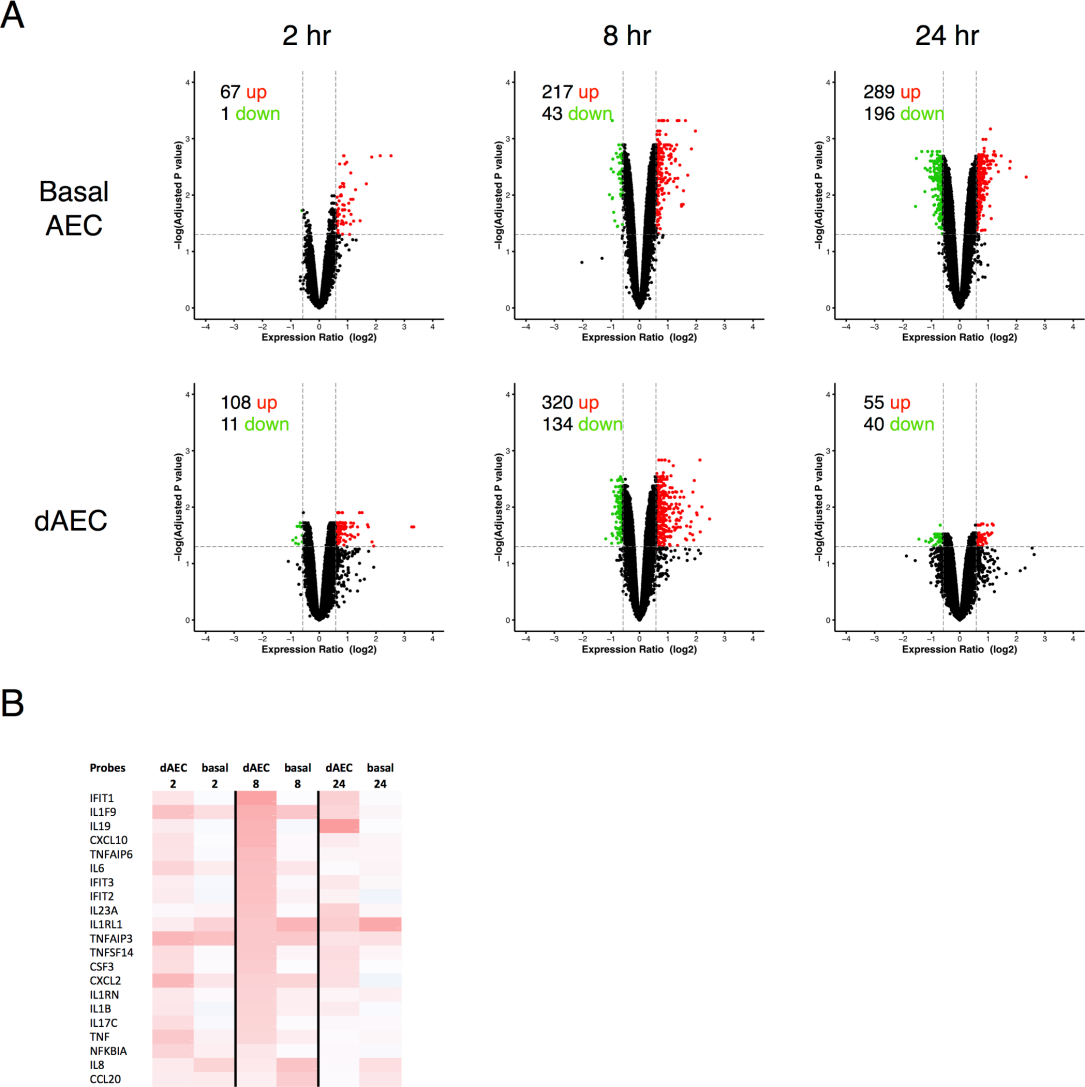
Normal basal and differentiated airway epithelial cells differ in gene expression patterns after mechanical injury. A. Volcano plots in AEC from asthmatic donor lungs to demonstrate differential expression in probes in the resting (without mechanical injury) state (N = 6 in each group) between differentiated (dAEC) and basal AEC, using all expressed gene probe sets (n = 41,733) as an input dataset. For each, the number of up- and down-regulated probes (≥1.5 fold change, vertical dashed lines, adjusted P < 0.05, horizontal dashed line) is provided. B. Differential expression for selected gene probes involved in inflammation and inflammation signaling at 2–24 hr after injury versus non-injured cells is provided as a heat map.

After the mechanical injury, both differentiated and basal AEC from asthmatic subjects expressed a limited, discrete set of up-regulated genes, and fewer down-regulated genes (Fig 4A). Furthermore, the expression of cytokine and chemokine ligands was similar in differentiated and basal AEC (Fig 4B). These patterns were consistent to the ones observed in normal AEC and dAEC.

**Fig 5:**
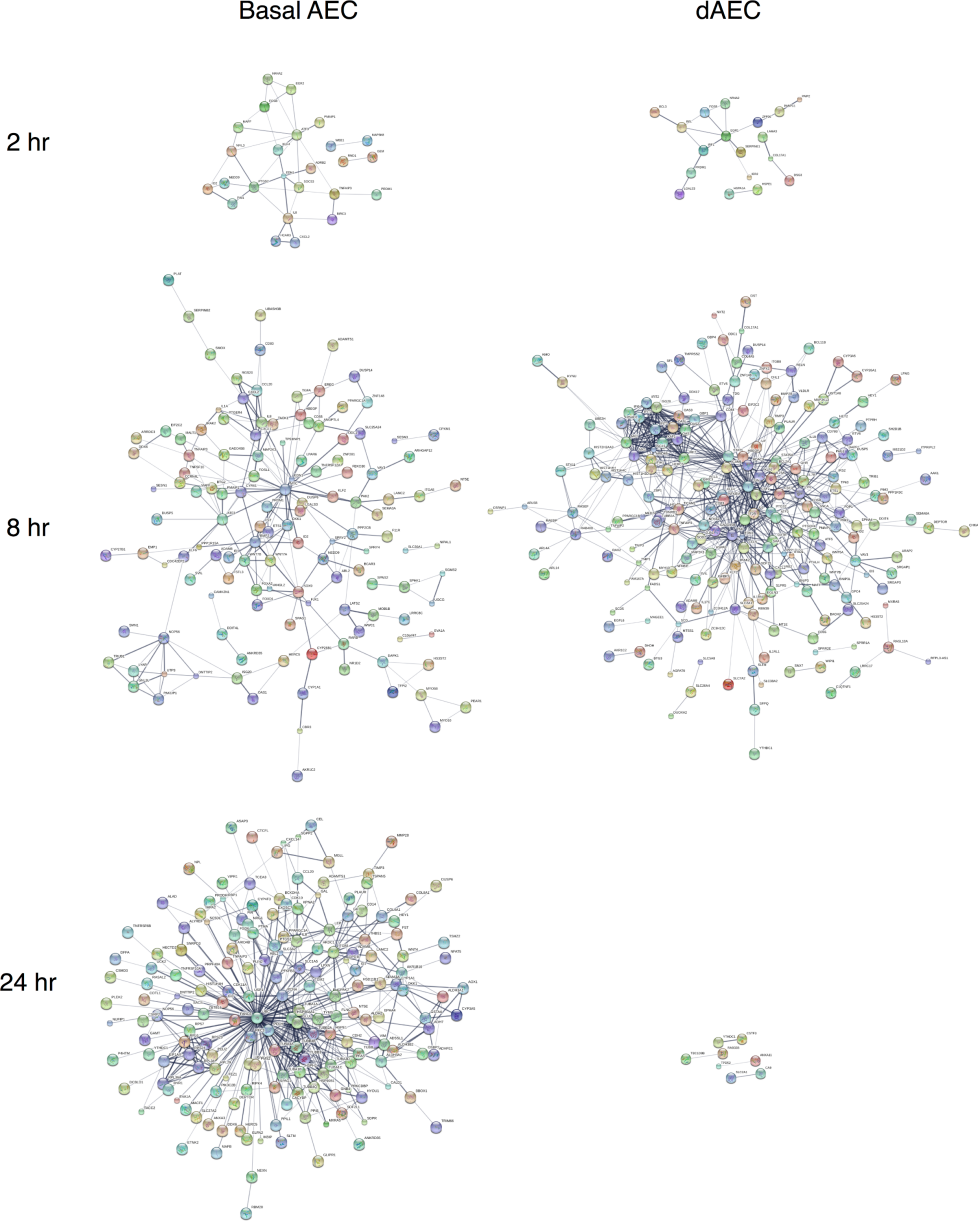
Reconstruction of molecular networks for asthmatic basal and differentiated AEC after mechanical injury. String networks generated from WGCNA modules using the genes in significant DE at 2–24 hr after injury for each cell type to demonstrate functional relationships in molecular networks. Highly connected network activation was similar at 2 hr, richer and more substantial in differentiated (dAEC) versus basal AEC at 8 hr, and less significant at 24 hr after MI in dAEC compared to basal AEC. Probe labels are as shown.

Reconstruction of the molecular networks for the significantly DE genes (p-value<0.05, fold change > 1.5) using the STRING10 server is presented in Fig 5. The connections in dAEC were more complex and denser at 8 hr (with 771 edges, 9.071 average degree) versus 2 hr (with 76 edges 4.75 average degree). There were decidedly few connections observed at 24 hr with only 6 edges, which fit with the clear change in cytokine/chemokine expression changes and the far fewer functional pathways identified at that time point.

WGCNA analysis for each condition only detected 1 or 2 significant modules (Table K in S1 File). In 8 hr, the significant module detected for dAEC culture shows enrichment for cytokine activity, inflammatory response, interferon signaling pathway, cell death, TNF pathway and toll-like receptors while in AEC culture the detected module is enriched for regulation of phosphorylation, cell death, and differentiation (Table L in S1 File).

### Epithelial transcriptomes in normal and asthmatic basal and dAEC differ after mechanical injury

**Fig 6:**
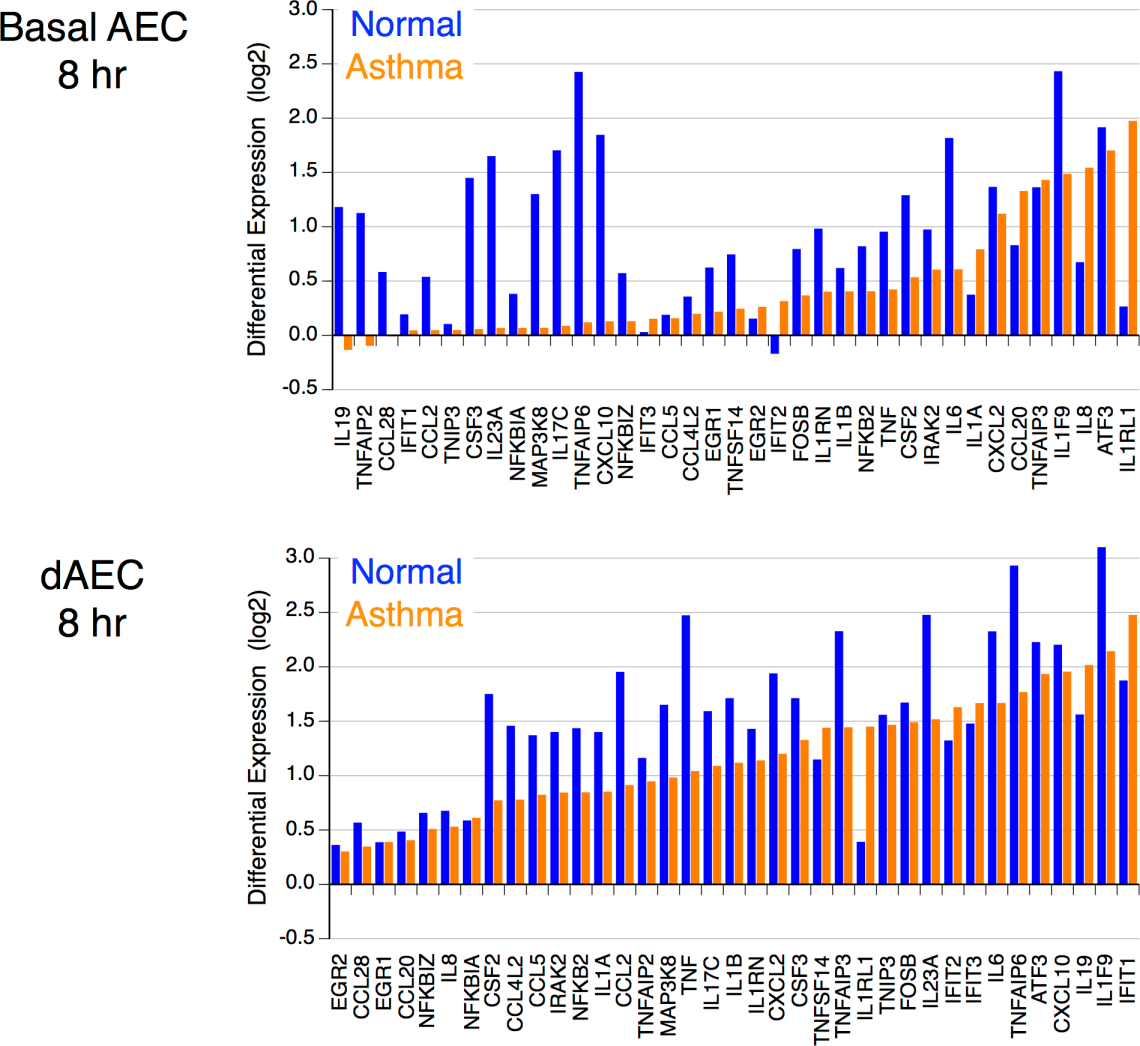
Comparison of differential gene expression for selected inflammatory and signaling genes in basal and differentiated AEC collected from normal and asthmatic donors. In each graph, expression is arranged in increasing expression in asthma AEC. Gene expression patterns differ between the two disease states for cytokines and chemokines. N = 4 in normal donors and 6 in asthmatic donors.

The analysis of the transcription in both differentiated dAEC and basal AEC after mechanical injury demonstrated significant differences for the cells collected from normal and asthmatic subjects at all time points after injury. These included variations in expression patterns of cytokines, chemokines and genes involved in related signaling pathways both in asthmatic basal and differentiated AEC. Fig 6 shows the gene DE between asthmatic and normal AEC, and dAEC at 8 hours after the injury. The expression of the genes involved in cytokine response to injury generally was lower in asthmatic epithelium both in AEC and dAEC. However, the expression of IL8, IL1A, IL1RL1, as well as CCL20 genes was higher in asthmatic basal AEC in comparison to normal basal AEC. The expression of IL-19, TNFSF14, as well as the NFKB-independent IL1RL1 interleukin-like receptor also was increased in asthmatic dAEC. These cells also demonstrated higher expression levels of the genes that encode the interferon-induced IFIT proteins (IFIT1, IFIT2, and IFIT3). The DE of key genes at each time point after MI are presented in Table M in S1 File. These data suggest a substantially different pattern of gene expression in asthmatic epithelial cells as compared to normal AEC.

### Enrichment analysis

The enrichment analysis of genes differentially expressed (p < 0.05, FC >1.5) at each time point has revealed the significant activation of functional categories related to transcription (GO:0008134), cytokine and chemokine activity (GO:0034097), inflammation, programmed cell death (GO:0012501), and to defense responses against bacteria both in basal and differentiated AEC (Table 2, basal AEC; and Table 3, differentiated AEC). Asthmatic AEC transcriptomes were enriched for the genes involved in the pathways related to transcription and cell death 2 to 8 hr after MI, and to cell cycle 24 hr after MI in comparison to functional pathways generated in normal AEC (Table 1). Asthmatic DE genes also contained fewer cytokine and chemokine genes.

**Table 2:** Selected functional genetic ontology (GO) pathways identified in asthmatic airway epithelial basal cells after mechanical injury in 8 hours.

GO term Name	P value	Adjusted P-value	In query set	In whole genome	Genes
GO:0012501 programmed cell death	4.6E-11	3.9E-08	51	1952	MYC,SOX9,GADD45B,SPRY2,ABL2,TP53INP1,TNFSF10,GAS1,ETS1,IRAK2,DAPK1,PPP1R15A,ITGA5,PHLDA2,ADAM8,F3,TNFRSF12A,SEMA3A,BMP2,PPARGC1A,MALT1,TSC22D1,BTG2,SPHK1,TAOK1,VAV3,PMAIP1,CYP26B1,RARB,PHLDA1,TSC22D3,WNT7A,WNT7B,G0S2,DUSP6,ID1,ID3,EDN1,LGALS3,FOSL1,TGFA,PPP2CB,LATS2,CYR61,ANGPTL4,SERPINB2,PAK2,ATF3,TNFAIP3,CKAP2,IL1A
GO:0042981 regulation of apoptotic process	5.4E-11	3.9E-08	44	1519	MYC,SOX9,GADD45B,SPRY2,ABL2,TP53INP1,TNFSF10,GAS1,ETS1,IRAK2,DAPK1,PPP1R15A,ITGA5,ADAM8,F3,TNFRSF12A,BMP2,PPARGC1A,MALT1,TSC22D1,BTG2,SPHK1,VAV3,PMAIP1,RARB,PHLDA1,TSC22D3,WNT7A,G0S2,DUSP6,ID1,ID3,EDN1,LGALS3,FOSL1,TGFA,LATS2,CYR61,ANGPTL4,SERPINB2,PAK2,ATF3,TNFAIP3,IL1A
GO:0060429 epithelium development	7.6E-11	4.2E-08	40	1296	MYC,SOX9,CYP1A1,SPRY2,MYADM,EREG,ABL2,CYP27B1,GAS1,ITGA5,FOXA2,AKR1C2,KLF2,PRDM1,SEMA3A,BMP2,F11R,UGCG,MAFF,FST,FOXD1,CYP26B1,RARB,HBEGF,WNT7A,WNT7B,ID1,ID2,ID3,LAMC2,EDN1,LGALS3,VANGL2,RGS20,LATS2,DKK1,CYR61,ARHGAP12,EMP1,IL1A
GO:0005154 epidermal growth factor receptor binding	1.5E-05	1.1E-02	5	33	EREG,ITGA5,VAV3,HBEGF,TGFA

**Table 3:** Selected functional genetic ontology (GO) pathways identified in asthmatic airway epithelial differentiated cells after mechanical injury in 8 hours.

Go term Name	P value	Adjusted P-value	In query set	In whole genome	Genes
GO:0034097 response to cytokine	1.9E-20	1.0E-16	59	825	ETS1,KLF4,KLF2,ZBP1,CCL5,FLRT3,FLRT2,HSPA5,VLDLR,ZC3H12A,KLF6,WNT5A,ICAM1,IFITM1,BIRC3,IFIT2,IFIT1,IFIT3,RSAD2,CSF3,ISG15,OASL,XAF1,SNCA,IL1RN,IFI6,IL6,MX1,IL7R,MX2,SPARC,TNFSF14,IRAK2,GBP1,IRF7,ISG20,PTGER4,PTGS2,STAT1,STAT5A,GHR,JUN,GBP4,NFKB2,NFKBIA,KYNU,TRIM31,OAS1,OAS2,OAS3,CXCL2,EDN1,OXTR,SERPINE1,CD44,TNF,IL1RL1,TNFAIP3,IL36G
GO:0006952 defense response	1.6E-19	4.5E-16	84	1651	TNIP3,ETS1,TNIP1,CD83,KLF4,ADAM8,F2R,S100A7,ZBP1,SAA1,ADRB2,HIST1H3D,SCD,CCL5,PMAIP1,IL19,DUOXA2,TPST1,DDIT4,ZC3H12A,WNT5A,ICAM1,IFITM1,BIRC3,SP110,IL23A,IFIT2,IFIT1,IFIT3,SLC7A2,RSAD2,ISG15,SH2D1B,OASL,PDE5A,XAF1,SNCA,STX11,IL1RN,MAP2K3,IFI6,IL6,MX1,MX2,HDAC9,ZNF148,TICAM1,IL17C,MTSS1,CD55,IRAK2,GBP1,IRF7,ISG20,BCL3,PTGER4,ZP3,PTGS2,STAT1,STAT5A,IFIH1,GJA1,BNIP3,BNIP3L,GBP4,NFKB2,NFKBIA,KYNU,NR1D2,TRIM31,OAS1,OAS2,OAS3,CXCL2,EDN1,P2RY11,SERPINE1,CD44,TNF,IL1RL1,TNFAIP3,LYN,IL36G,HERC5
GO:0009607 response to biotic stimulus	1.9E-18	2.1E-15	63	1027	TNIP3,F2R,S100A7,ZBP1,HIST1H3D,SCD,CCL5,HSPA5,PMAIP1,VLDLR,DDIT4,ZC3H12A,WNT5A,ICAM1,IFI44,IFITM1,BIRC3,SP110,IL23A,IFIT2,IFIT1,IFIT3,IVNS1ABP,RSAD2,CSF2,CSF3,ISG15,OASL,SNCA,IL6,MX1,MX2,SPARC,TICAM1,MTSS1,CD55,IRAK2,GBP1,IRF7,ISG20,BCL3,PTGER4,PTGS2,STAT1,IFIH1,JUN,BNIP3,BNIP3L,GBP4,NFKB2,NFKBIA,OAS1,OAS2,OAS3,ODC1,CXCL2,EDN1,SERPINE1,TNF,TNFAIP3,LYN,TRIB1,HERC5
GO:0060337 type I interferon signaling pathway	1.6E-16	1.2E-13	19	81	ZBP1,WNT5A,IFITM1,IFIT2,IFIT1,IFIT3,RSAD2,ISG15,OASL,XAF1,IFI6,MX1,MX2,IRF7,ISG20,STAT1,OAS1,OAS2,OAS3
GO:0005125 cytokine activity	3.8E-07	1.2E-04	17	222	CCL5,IL19,GDF15,WNT5A,IL23A,CSF2,CSF3,IL1RN,IL6,TNFSF14,IL17C,CXCL2,EDN1,LIF,TNF,CMTM7,IL36G
GO:0005126 cytokine receptor binding	1.4E-05	3.3E-03	17	289	TGFBRAP1,CCL5,GDF15,IL23A,CSF2,CSF3,IL1RN,IL6,TNFSF14,IL17C,STAT1,CXCL2,LIF,CD44,TNF,LYN,IL36G
GO:0070851 growth factor receptor binding	4.2E-05	8.1E-03	11	143	VAV3,FLRT3,FLRT2,CSF2,CSF3,IL1RN,IL6,HBEGF,CD44,LYN,IL36G

**Fig 7:**
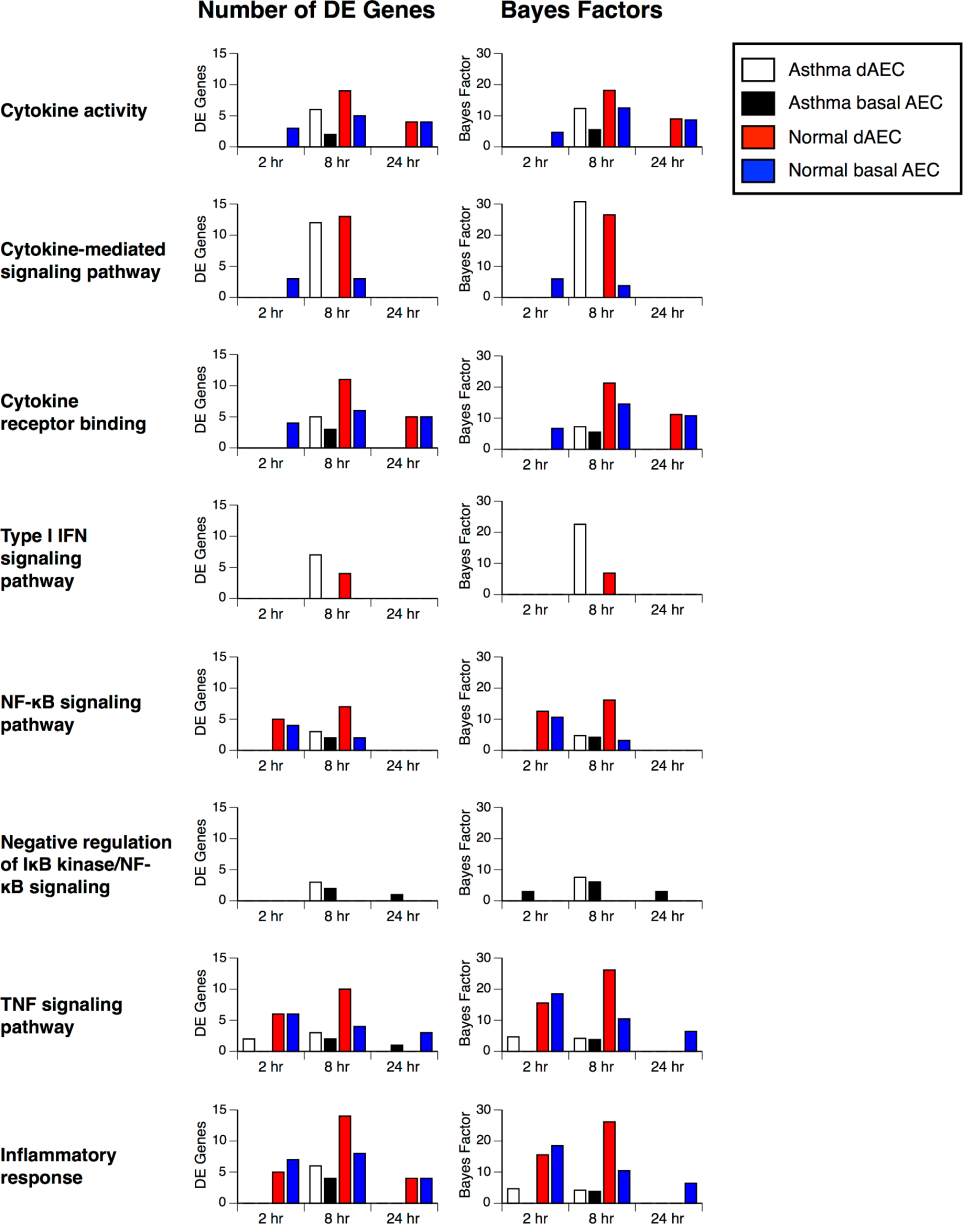
Increased differential expression of genes in select inflammatory functional pathways after mechanical injury. The number of expressed genes and calculated Bayes factors from enrichment analysis for each functional category in differentiated (dAEC) and basal AEC from normal and asthmatic airways. There are more expressed genes in normal versus asthmatic cells, and differentiated versus basal cells, in most categories.

Examination of functional pathways based on the number of DE genes, confirmed the initial analysis of gene DE enrichment analysis. At each time point in many of the functional pathways, AEC from normal donors had a greater number of DE genes and higher Bayes factors compared to AEC from asthmatic donors, and differentiated cells had a greater number of DE genes compared to basal AEC (Fig 7).

## Discussion

An important issue in tissue repair after airway mucosal injury is to define the mechanisms by which inflammatory factors, growth factors, and other effectors influence the renewal and differentiation of resident cells. Especially important are early events after injury that initiate or perpetuate inflammatory responses in the immediate and neighboring regions. Because multiple factors are produced in the first hours after injury, it is critical to understand the initial responses of cells so as to determine whether these signals, alone and in combination, serve as pro- or anti-reparative signals or as autocrine or paracrine signals for repair and inflammation in neighboring cells and tissue. While many studies to date have evaluated the role of an individual protein or a handful of factors in elegant ‘reductionist’ models to illustrate their key roles in these processes, few have examined the orchestrated response of epithelial cells, particularly in airways, after discrete injury. These orchestrated responses, from transcriptional to post-translational, serve to coordinate reparative and inflammatory signals that are appropriate in normal responses to injury, and that may become aberrant in disease states depending on context.

We have examined the AEC transcriptome in the first hours after injury with an emphasis on the expression of cytokines and chemokines that can influence inflammation in the airway mucosa and submucosa, particularly that with identified importance in asthma. We identified a distinct gene expression profile of such mediators in both differentiated and basal normal AEC that left unchecked would contribute to inflammatory cell influx into the mucosa. Differentiated AEC demonstrated a richer gene expression pathways of pro-inflammatory chemokines than basal AEC, suggesting that a normal, homeostatic airway epithelium is more capable of responding to injury with pro-inflammatory mediator expression. Basal AEC have a central role in repair after injury in airways, from the initial steps of spreading and migration to later proliferation and phenotype shifting into needed new cell subtypes [1, 2, 4, 42, 43]. In contrast to the response seen in differentiated AEC, these pluripotent basal cells were less capable of expressing key cytokines and chemokines. Gene expression networks relevant to the cytokines response differed significantly in the first 24 hr after injury. In an airway with chronic damage to or loss of ciliated and goblet cells, as is frequently encountered in severe asthma, basal AEC are more dominant, and thus the pro- and anti-inflammatory responses signaled by them may differ from that seen with an intact, differentiated epithelial layer. These observations have clear implications for the responses of the neighboring structural and inflammatory cells and may become magnified over time with repeated cycles of injury and (aberrant) repair.

**Fig 8:**
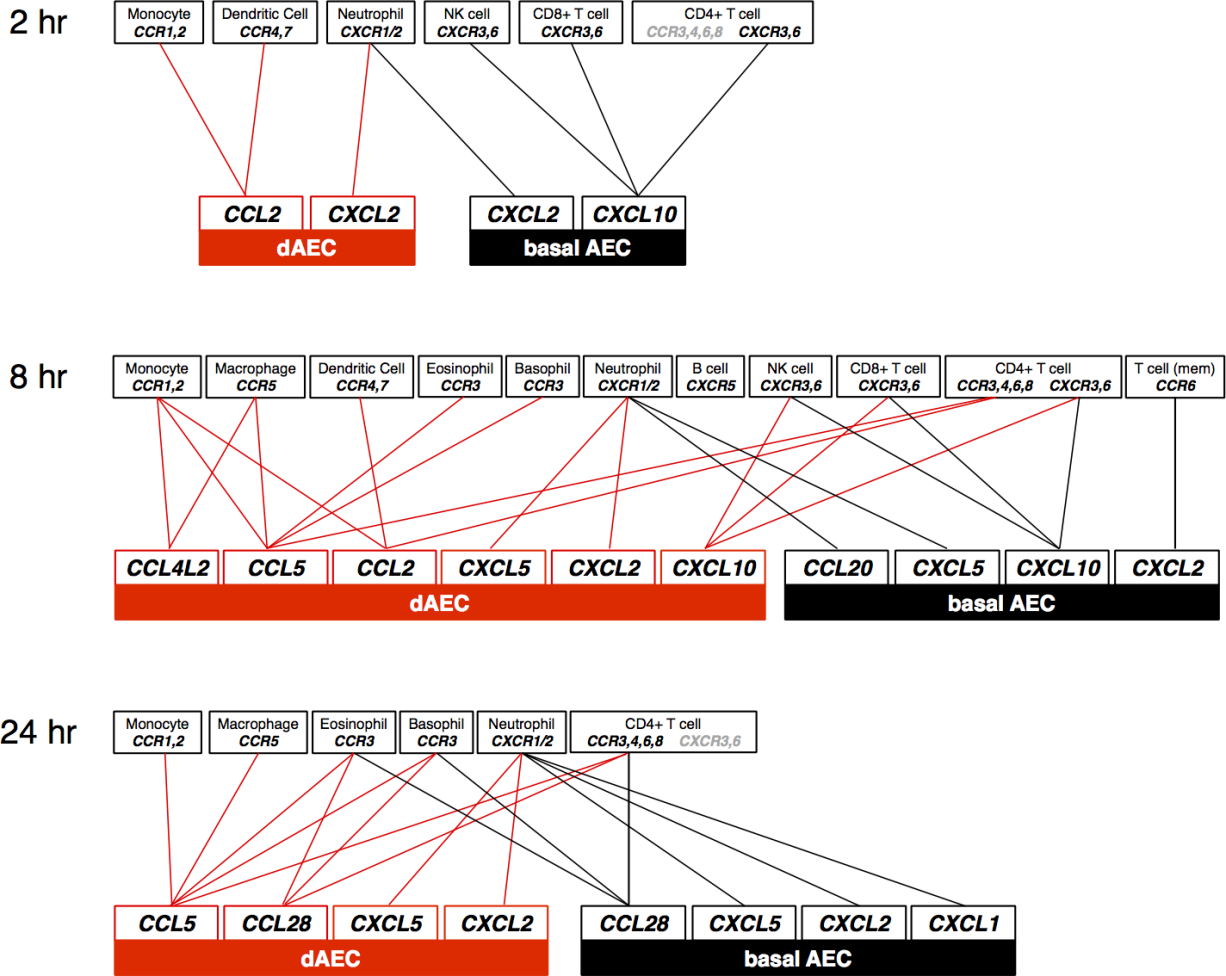
Expression of genes clustered for other physiologic processes important to the epithelial reparative response in the first 24 hr after MI such as cell cycle and proliferation, migration, and differentiation. Heat maps of these time points and conditions demonstrated few genes in DE.

Several classic inflammatory cytokines such as IL-6, IL-8, and TNF-α, and signaling intermediate genes common to these receptors in the NF-κB and TNF-α pathways, were increased in each cell set, particularly 2 and 8 hr after injury. The recently described IL1F9 (IL-36γ), a ligand for a heterodimeric receptor consisting of IL-1R2 and IL-1RAP subunits that can activate NF-κB, extracellular signal-regulated kinase (ERK) and c-Jun-N-terminal kinase (JNK) signaling pathways [44], also was consistently up-regulated at each time point. Other cytokine and chemokine ligands with important roles in inflammatory cell recruitment demonstrated a different pattern with increased expression in dAEC compared to basal cells: these included CCL5, CCL2, CXCL2, and CCL4L2. CCL5, also known as Regulated upon Activation, Normal T-cell Expressed, and Secreted (RANTES), is the ligand for receptors CCR1, CCR3, and CCR5 [45], and its greater expression in cells in dAEC after MI suggests the potential for recruitment of monocytes, eosinophils, basophils, T cells, and dendritic cells. Likewise, CCL2, also known as monocyte chemoattractant protein-1 (MCP-1), is a ligand for CCR2 on monocytes [46, 47] and for CCR4 and CCR7 on dendritic cells [48, 49]. CCL4L2, a ligand for CCR1 and CCR5 on macrophages and monocytes, is a polymorphism of CCL4L1, itself a non-allelic copy of CCL4 that differs in a single amino acid [50] and all of which are associated with psoriasis severity [51]. The CCL4L2 activity has not been described previously in airway epithelium, but demonstrated increased expression in dAEC 8 hr after injury in the current study. The increased expression of CXCL2 (also MIP-2 or GRO-β), an IL-8 homolog and ligand for CXCR-2 that mediates neutrophil recruitment to the lung [52, 53], and CXCL10 (ligand for CXCR3), also suggested potential for the recruitment of multiple inflammatory cell types [54–56]. A mapping of these cytokine and chemokine ligands to the potential inflammatory cell receptors is shown in Fig 8. The DE of IL-17C, a key mucosal host defense cytokine produced in airway epithelium [57] which binds the IL-17RE-IL-17RA receptor complex [58, 59], was in significantly increased at 2 and 8 hr after MI in normal but not asthmatic cells. These data suggest that a more robust mucosal host defense can be elicited early after injury in normal AEC.

Of interest is the observation that more differentiated cells have a richer DE early (2 and 8 hr) after the injury reflected by the variety of functional categories in enrichment analysis, than basal cells. Dissecting this difference is hampered due to the fact that the differentiated ciliated and goblet columnar cells require a mixed culture in which basal cells provide for anchoring and survival and cannot be isolated and grown in pure culture. The relative contribution of each cell type then is not clear from our findings, but may become important in models of chronic airway inflammation in which goblet cell metaplasia is prominent [60–63].

## Conclusions

Asthmatic airway inflammation frequently is initiated by injury to the epithelial cells lining central airways. Gene expression is essential for orchestrating the early response to epithelial cell injury. We report that basal and differentiated airway epithelial cells collected from asthmatic lungs have diminished immediate cytokine and chemokine response to injury in comparison to non-asthmatic, normal AEC and dAEC. Gene expression in asthmatic cells instead is centered on reparative processes including proliferation, cell cycle, and differentiation. While airway inflammation is the *sine qua non* of asthma, intrinsic dysfunction of the asthmatic airway epithelium as demonstrated by decreased expression of inflammatory pathways after injury suggests that paracrine factors are required to augment epithelial-derived inflammatory responses after injury.

## Supporting information

S1 Letter Letter from the University of Chicago institutional review board. (PDF)

S1 File Single file that contains supporting information Tables A–M.
(XLSX)
