# Supplementary material for: Chemokine expression in the early response to injury in human airway epithelial cells
Source: PLoS One. 2018 Mar 13;13(3):e0193334. doi: 10.1371/journal.pone.0193334 (PMC5849294; doi:10.1371/journal.pone.0193334)
Supplement: S1 Letter — (PDF) [file pone.0193334.s001.pdf]

The Division of Biological Sciences • The Pritzker School of Medicine
The University of Chicago Hospitals

Institutional Review Board
McGiffert Hall, 2nd Floor
5751 S. Woodlawn Ave.
Chicago, Illinois 60637
(773) 702-6505

Date: March 12, 2010

To: JULIAN SOLWAY

From: BSD/UCH Institutional Review Board

Re: 11122A "Smooth muscle abundance in lung"

Funding Source(s):
NIH (Mechanisms of Force Fluctuation-Induced Relenthening in Airway Smooth: TRACS ID 32487)
Astrazeneca (The influence of Budesonide and Flormoterol on Force Fluctuation-Induced Relenghening of contracted airway smooth muscle: TRACS ID 36610)

A representative of the IRB has reviewed the above referenced submission, which outlines to characterize the biochemical nature of smooth muscle in human airway tissue and to obtain tissue for isolation, culture, and functional evaluation of airway smooth muscle. You indicate that "tissue is obtained from donor lungs that could not be used for transplantation or from surgical or autopsy specimens that otherwise would have been discarded". You have further indicated that you no samples will be obtained from any living subject.

As per the Office of Human Research Protection regulation, 45 CFR46.102 (f), human subjects are defined as "living individuals(s) about whom an investigator (whether professional or student) conducting research obtains (1) data through intervention or interaction with the individual, or (2) identifiable private information." As you will not be obtaining any information about or samples from living individuals, this research does not constitute engagement in human subjects research. Therefore your study does not require review by the IRB. Please note that in the event you or your research team at the University of Chicago alter this research to include any living individuals as subjects, the research activity would then involve human subjects under the DHHS regulations. In addition, please note that if you are listed as key personnel on a grant funding this project, stipulations of the grant regarding access to data may require that a proposal be submitted to the IRB. Please contact the IRB for guidance if such an occasion should arise. Thank you for bringing this matter to the attention of the IRB. You may proceed with this project.
